# The Role of Health Claims on Consumer Behavior and Food Choice: A Narrative Review

**DOI:** 10.3390/foods15040773

**Published:** 2026-02-20

**Authors:** Helena F. Martins Tavares, Geni Rodrigues Sampaio, Adriano Costa de Camargo, Elizabeth Aparecida Ferraz da Silva Torres

**Affiliations:** 1 Department of Nutrition, School of Public Health, University of São Paulo, Av. Dr. Arnaldo 715, São Paulo 01246-904, SP, Brazil; nutri.helenatavares@gmail.com (H.F.M.T.); genirs@usp.br (G.R.S.); 2 Instituto de Ciencias Aplicadas, Universidad Autónoma de Chile, Santiago 7500910, Chile

**Keywords:** health claim, functional food, consumer behavior, decision-making, purchase intention

## Abstract

Data suggests that consumers are increasingly aware of the nutritional composition of foods, and the presence of health claims is considered a differentiating factor in the purchase of foods and beverages. We analyzed literature on health claims and their impact on consumer behavior, using different databases (Web of Science, Scopus, and PubMed). According to the bibliometric analyses of 423 articles, research on health claims presents distinct subareas such as health, marketing, regulation, public health, and behavior. Data from several studies, involving 27,813 participants from several countries, are summarized. The health claims included: cardiovascular, bone, muscle, metabolic, digestive, eye, along with overall health and wellness. Antioxidant and anti-inflammatory properties, cognitive and mental performance, immune system support, and disease prevention were also addressed. Our resulting narrative review indicates that health claims could have a positive influence on consumer behavior, especially about the perception of value, purchase intention, and willingness to pay for foods that feature this type of communication. Although health claims on foods have a significant potential to positively influence consumer-purchasing behavior, their impact is dependent on multiple individual and contextual factors, such as consumers’ health status and knowledge on nutrition, price, taste, access, and consumers’ perception of the brand. Understanding the relationship between health claims and consumer behavior and choices is essential to developing effective regulations, public policies, and communication strategies to encourage healthier food choices and influence the food industry.

## 1. Introduction

The food industry is responsible for a major part of modern production and consumption. By 2024, the global food market was projected to be worth $9.12 trillion, growing at an annual rate of 6.7% according to the World Economic Forum [1]. Consumers are more informed about the production, processing, and distribution of food, paying close attention to nutritional content, environmental impact, and social responsibility of companies within the food supply chain. Their growing interest in the nutritional qualities of food is now a major driver of research and innovation in food development [2,3]. The consumer awareness on the relationship between food and health has created a market for functional foods that explore nutritional properties using health claims [4,5], and this has been reinforced by the new trends in nutrition due to the recognition of the WHO on the importance of GLP-1 agonists that bring together new challenges for the health and food industry [6]. The demand for functional foods and beverages is driven by rising healthcare costs, increasing life expectancy, which has also been suggested as a possible outcome of GLP-1 therapy [7], and a growing desire for a better quality of life. In this context, functional foods are important, providing a category of products that deliver targeted health benefits through specific ingredients [8]. The development of functional foods entails an innovation process across multiple stages from the initial innovative concept to market launch, production, distribution, and the effective communication of benefits through health claims. Communicating these benefits requires extensive research into the food’s health effects, followed by a regulatory review before the product reaches consumers. This regulatory process involves translational science and depends on peer-reviewed studies that demonstrate the efficacy and safety of the compounds (nutrients, probiotic or bioactive substances) within the food matrix or supplement. These studies must comply with each country’s regulations to gain approval and enable commercialization [9]. Once approved, health claims may appear on food labels, promotional materials, and other advertising platforms. Usually, countries present strict regulations on health claims, resulting in challenges for the approval and communication of health-related messages in functional foods.

The first functional food regulation, called “Foods for Specified Health Uses” (FOSHU), was introduced in Japan in 1991 by the Ministry of Health, Labor and Welfare [10]. After the introduction of the FOSHU regulation, the number of functional food products increased, especially between 1997 and 2007, as consumer demand grew. The net sales of FOSHU products reached their peak in 2007 at 6.2 billion USD. About half of the health claims were related to improving gastrointestinal tract health by probiotic lactobacilli, oligosaccharides, and dietary fiber. Around 20% of the products carried health claims linked to reductions in serum triglycerides. Another 20% of the products featured health claims addressing high blood pressure, elevated LDL-cholesterol, high blood glucose, tooth decay, and mineral absorption. Regulatory approaches to health claims vary internationally but share common principles. Since the introduction of FOSHU, health-claim regulations have evolved to balance innovation with consumer protection [10,11]. Similar frameworks have been established globally, including those adopted by Codex Alimentarius, the European Union, the United States, Canada, and Australia/New Zealand, which generally distinguish between different categories of claims—each requiring specific levels of scientific substantiation [12,13,14,15,16]. Despite regulatory convergence in definitions and objectives, differences in claim categories and approval processes add complexity to how health-related information is communicated to consumers.

Internationally, the concept of “health claims” is generally broad and encompasses different subtypes, such as function and structure claims and disease risk reduction claims, each following distinct regulatory requirements and levels of scientific substantiation.

In accordance with the Codex Alimentarius and the European Union, a health claim means any representation that states, suggests, or implies that a relationship exists between a food or a constituent of that food and health. Health claims include the following: (i) nutrient function claims: that describes the physiological role of the nutrient in growth, development and normal functions of the body (example: “Calcium is needed for the maintenance of normal bones”); (ii) function claims: claims relate to a positive contribution to health or to the improvement of a function or to modifying or preserving health (example: “Olive oil polyphenols contribute to the protection of blood lipids from oxidative stress”); (iii) reduction in disease risk claims: claims relating to the consumption of a food or food constituent, in the context of the total diet, to the reduced risk of developing a disease or health-related condition (example: “Plant sterols/stanol esters have been shown to lower/reduce blood cholesterol. High cholesterol is a risk factor in the development of coronary heart disease”).

For consistency throughout the study, the term “health claims” is used as an umbrella concept including different regulatory subcategories, including function and disease risk reduction claims. The term “functional claims” is used only when referring specifically to claims related to the functional effects of foods or food constituents. In addition, “consumer behavior” is employed as a broad construct that includes attitudes, food choice, purchase intention, and decision-making processes related to foods bearing health claims. In theory, health claims are intended to support informed consumer behavior. However, their effectiveness depends on consumers’ ability to comprehend, trust, and appropriately use the information provided.

Previous studies have investigated consumer behavior toward health claims, including their understanding of claim wording [17]. Nevertheless, findings across studies remain fragmented, influenced by factors such as claim type [18], product category [19], individual characteristics [20], and contextual cues [21,22,23,24]. Moreover, despite the growing volume of research on health claims, no bibliometric analyses have been carried out that systematically map how scientific production has addressed the relationship between health claims and consumer behavior. This gap limits a comprehensive understanding of how the field has evolved and where research efforts have been concentrated.

To address these gaps, our contribution combines a bibliometric analysis with a narrative review to (i) quantitatively map international scientific evidence on health claims and their role in consumer behavior and (ii) qualitatively synthesize recent evidence on how health claims influence consumer decision-making, particularly considering emerging nutritional trends. By integrating bibliometric mapping with a narrative review, our study provides a novel and structured overview of the scientific landscape of the impacts of health claims on consumer behavior, while also offering a qualitative synthesis of recent evidence. Our dual approach identifies research trends and knowledge clusters as well as interprets how different types of claims influence consumer behavior. The findings contribute to academia, regulatory agencies, and the food industry.

## 2. Materials and Methods

**Figure 1:**
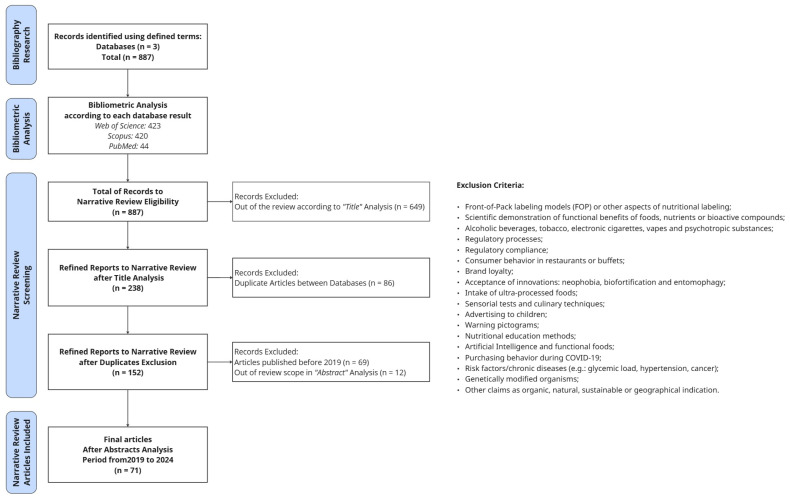
Methodology Diagram. Based on PRISMA.

The methodological approach of this study is detailed in Figure 1 and comprises two complementary and sequential stages: a bibliometric analysis followed by a narrative review, each with distinct objectives and inclusion criteria. This sequential approach ensured methodological transparency while allowing both a broad quantitative mapping (bibliometric analysis) and a qualitative interpretation of the literature (narrative review).

### 2.1. Bibliometric Analysis

The bibliometric analysis aimed to provide a quantitative overview on the evolution of the scientific evidence linking health claims to consumer behavior. For this purpose, to summarize the evolution in the field, regardless of the year, all articles retrieved from the Web of Science, Scopus, and PubMed databases were included. No exclusion criteria were employed at this stage. The bibliometric analysis focused on publication trends, keywords, and citation patterns, using VOSviewer software 1.6.20 and graphical tools from the Web of Science database. The search was performed using the terms (including plurals): “health claim” or “functional claim” or “functional food” (All Fields) AND “attitude” or “food choice” or “consumer choice” or “consumer behaviour” or “consumer behavior” or “consumer decision” or “purchase behaviour” or “purchase behavior” or “purchase intention” (All Fields).

### 2.2. Narrative Review

This narrative review was designed to qualitatively synthesize recent evidence on how health claims and consumer behavior are associated. Different from the bibliometric analysis, for the narrative review, predefined inclusion and exclusion criteria were employed (Figure 1). A multi-step screening process was conducted to refine the articles for inclusion in the narrative review, involving title and abstract screening followed by full-text assessment. Studies were excluded when their primary focus did not align with consumer behavior related to health claims. For example, front-of-pack nutrients labeling models, sensorial tests, regulatory approval evidence, brand loyalty, advertising to children, and other claims as “organic”, “natural”, or “sustainable” were excluded. Open-access articles published in English between 2019 and 2024 were considered. This temporal window was defined based on the bibliometric results, which indicated a marked increase and peak in publications from 2020 onwards, more precisely, in the years 2020 and 2022.

## 3. Results

### 3.1. Bibliometric Analysis

The bibliographic research identified a total of 887 articles, being 423 titles in Web of Science, 420 titles in Scopus, and 44 titles in PubMed. Table 1 exhibits data on articles retrieved from databases. The Web of Science and Scopus presented higher occurrences of articles related to claims and consumer behavior, compared with occurrences in PubMed. It was possible to identify a concentration of publications between 2019 and 2020. During the bibliographic research, there were no bibliometric analyses identified on this theme published. Therefore, the present analysis, as a quantitative analysis technique for scientific production, becomes important to help identify trends, assess the relevance of topics, and map knowledge networks.

#### 3.1.1. Areas of the Science Publishing About Health Claims and Consumer Behavior

**Figure 2:**
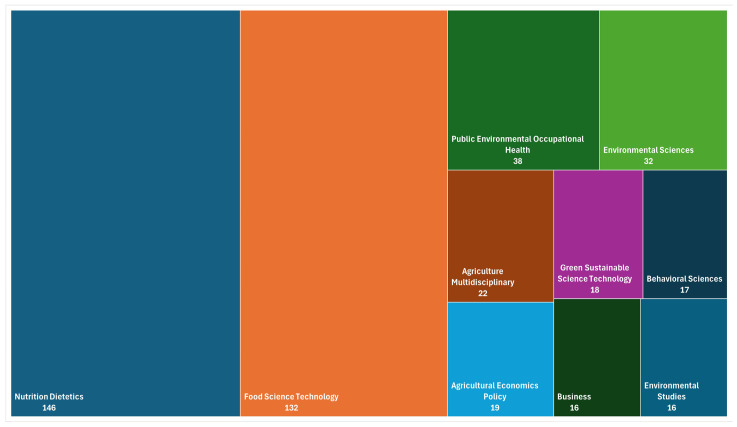
Areas of science are publishing about health claims and consumer behavior. Source: Web of Science.

According to Web of Science data in Figure 2, it is possible to observe the 10 main areas where publications about the theme are observed. Special attention to areas of Nutrition Dietetics (146 articles), Food Science Technology (132 articles), and only 17 articles in the Behavioral Sciences area.

#### 3.1.2. Countries, Citation Network, and Co-Authorship

**Figure 3:**
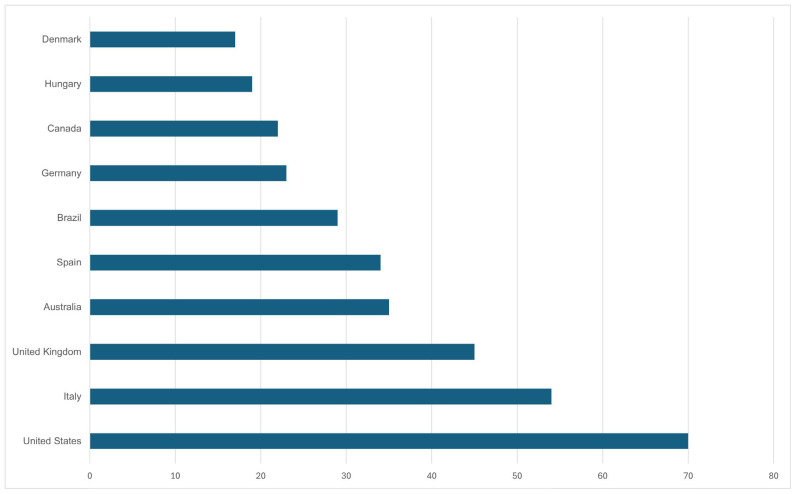
Countries with a high level of productivity on the theme. Source: Scopus.

**Figure 4:**
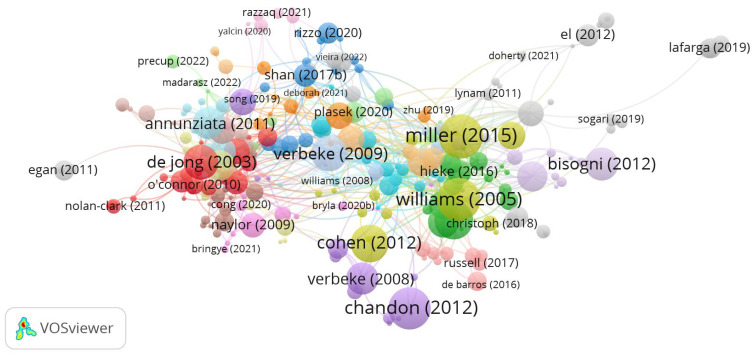
Citation network diagram from Web of Science. Source: VOSviewer.

Considering the analysis provided by Scopus (Figure 3), we identified 10 countries with high levels of productivity on the topic. Considering the most productive countries as Denmark, Hungary, Canada, and Germany, followed by Brazil. Exporting the Web of Science results to the VOSviewer system, it was possible to create a citation network diagram (Figure 4). This citation diagram visualizes the relationship between authors and their articles in terms of how often they are cited together. The different color groups (e.g., red, blue, green) represent clusters of authors who are frequently cited together. Red cluster may indicate an older line of research, possibly focused on established foundations or theories. A clear green cluster appears to be a group of authors who are frequently cited in a more contemporary context and may be related to more recent or emerging research. The size of the nodes (circles) indicates how often the author has been cited. The proximity between the nodes indicates how often these authors are cited together.

Close authors have a higher level of co-citation, meaning they are frequently discussed together in the literature. Some authors, such as Lafarga [25] and Egan [26], are further away from the main network. This suggests that their publications may be less connected to the central core of the research analyzed, possibly indicating a niche or less explored area. The years in parentheses after the authors’ names indicate the date of publication. It is possible to observe that there is a mix of older and recent publications, demonstrating the evolution of the area over time. This citation analysis allows the understanding of the main influences and how different research is interconnected. Central authors such as Miller [27] and Verbeke [28] are pillars, indicating works with great influence and impact. In Table 2, follow the list of the most cited authors by Web of Science results.

**Figure 5:**
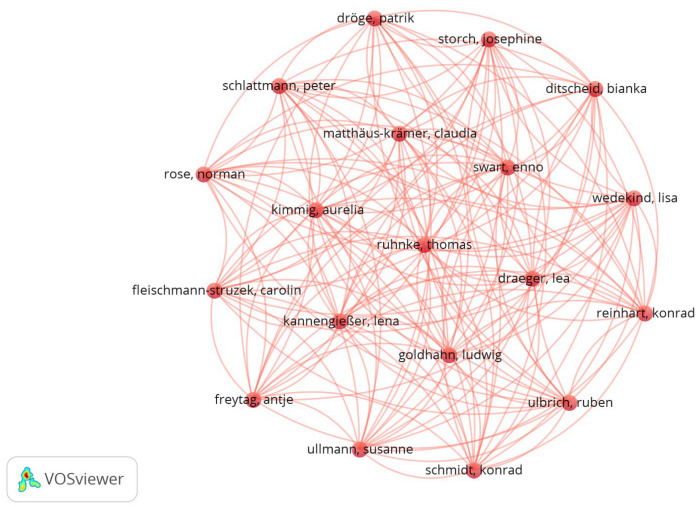
Full counting analysis for co-authorship from Scopus. Source: VOSviewer.

Considering the data resulting from Scopus, when exported to VOSviewer, a full counting analysis was created for co-authorship between the authors. Figure 5 shows a co-authorship concentration between 19 authors (from a total of 59 authors identified), with an important position of Ruhnke, Thomas, in a central position, indicating his high level of connections with the other authors. The same analysis was made using PubMed, and it showed a co-authorship concentration between 26 authors (from a total of 210).

**Figure 6:**
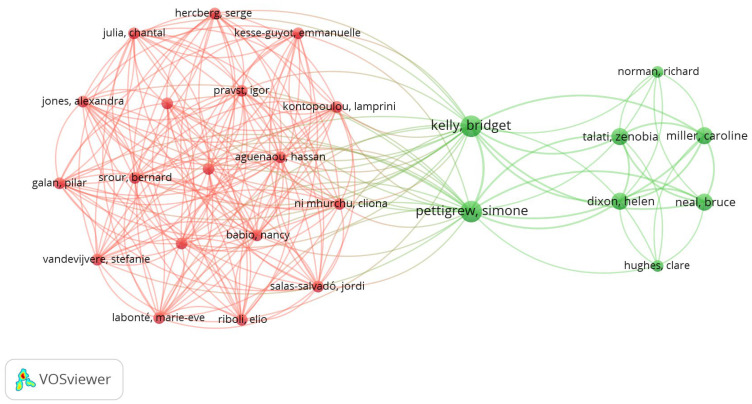
Full counting analysis for co-authorship from PubMed. Source: VOSviewer.

Figure 6 demonstrates that the authors on the left (red) represent a dense group of authors who appear to collaborate frequently with each other. The authors on the right (green) represent another cohesive group of authors with their own collaboration network, less connected to the red group. The central authors and those with larger nodes in the green group are the most influential or productive within their networks. The existence of some lines connecting the red group to the green group suggests that there is a small number of collaborative publications involving authors from both groups. The two groups (red and green) probably represent two distinct areas or subareas, with few points of intersection. Although there is interaction between the two clusters, collaboration seems limited. This may represent an opportunity to strengthen interdisciplinary collaborations to better integrate the knowledge of these networks.

**Figure 7:**
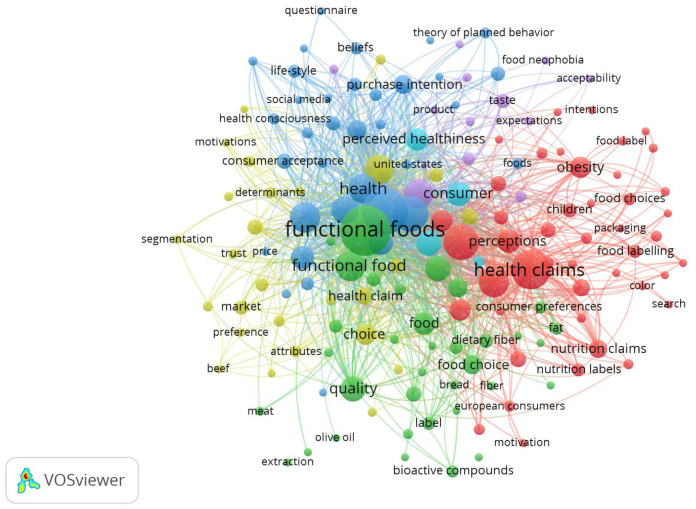
Keyword analysis from the Web of Science. Source: VOSviewer.

In the VOSviewer keyword analysis of the Web of Science results presented in Figure 7, it is possible to observe that larger words, such as “functional foods” and “health claims”, represent more frequent terms in the publications. This indicates that these are the central concepts or the most discussed in the research area. The colored clusters represent related topics grouped by the co-occurrence of the keywords in the documents, with each color identifying a group of terms frequently used together, indicating a subtheme or approach. The green cluster has terms such as “functional foods”, “food”, “quality”, “fiber”, “bread”, and “fat”, indicating this cluster may be related to studies that address behaviors based on the qualities of foods and their impact on health. The red cluster relates “health claims” with “food choices”, “nutrition labels”, “nutrition claims”, “perceptions”, “packaging”, “color”, “children”, and “obesity”. This cluster may reflect the discussion on nutritional labeling, food choices, and concerns related to obesity, advertising to children, and consumer preferences. The blue cluster includes words such as “perceived healthiness”, “purchase intention”, “theory of planned behavior”, “beliefs”, “acceptance”, and “consciousness”. This cluster highlights studies on purchasing behavior, consumer intention, and perception of health related to functional foods, which likely compose the group of refined studies for the elaboration of the narrative review. The yellow cluster connects to terms such as “trust”, “market, price”, “motivations”, and “segmentation”. This group is linked to market factors, such as consumer trust, price, and market segmentation for functional foods. The purple cluster links terms such as “taste”, “acceptability”, “expectations”, and “food neophobia”, indicating publications that could explore consumer behavior in the face of new foods and ingredients. The bibliometric review demonstrated that the research on functional foods is divided into distinct subareas encompassing health, marketing, regulation, public health, and behavior.

### 3.2. Narrative Review

A narrative review was conducted encompassing 71 articles from databases published from 2019 until 2024, with the exclusion criteria stated in Figure 1. The studies identified heterogeneous characteristics referring to populations, countries, products, and types of claims. Nevertheless, an overall positive role of health claims on food labels on consumer behavior is observed (Table 3).

Table 4 details examples from the Health Claims relationship with consumer behavior. Some health claims: cardiovascular, bone, muscle, metabolic, digestive, eye, as well as overall health and wellness. Antioxidant and anti-inflammatory effects, cognitive and mental performance, immune system support, and disease prevention were also addressed.

## 4. Discussion

### 4.1. Health Claims Relationship with Consumer Behavior

The analysis showed, in general, that health claims have a positive relationship with consumer behavior, especially with regard to the perception of value, purchase intention, and willingness to pay for foods that feature this type of communication [10,34,38,42,47,48,51,54,73,78,79,84,93,94]. However, it was observed that the magnitude of this influence is not homogeneous among consumers, being modulated by individual variables such as education, health consciousness, and nutritional knowledge [35,36,40,41,50,52,56,58,64,72,92]. Variables such as income, age, gender, product price, taste, convenience, emotions, preferences, concern about health, and desire to eat certain foods are also mentioned as modulators of this influence [2,37,52,55,60,61,65,67,68,89]. The choice of products carrying a claim was related to an increased perceived healthiness, health interest in food, and a sense of reward associated with nutritional health claims. Furthermore, consumers who are concerned about weight or are high in compensatory beliefs may be especially responsive to nutritional claims, while nutritional knowledge may act as a barrier against potential misleading claims [19].

A critical comparison of the reviewed studies reveals a contrast between those reporting relatively strong claim effects and those indicating limited or inconsistent impacts. In general, experimental and survey-based studies focusing on attitudes or purchase intentions often report positive responses to health claims, particularly when combined with salient peripheral cues such as packaging color or symbolic health attributes. In contrast, systematic and qualitative reviews highlight that such effects are frequently attenuated, context-dependent, or even questioned by consumers, especially when claim comprehension is low or skepticism toward marketing messages is high. This divergence suggests that health claims may enhance favorable perceptions under specific conditions, while their capacity to shape actual food choice remains limited.

According to Stuthridge et al. [20], despite the abundance of nutrition content and health claims on food labels, most consumers perceived these as a marketing tool and did not report consciously using them in food purchasing decisions. In addition, confusion is due to difficulties interpreting the claims with other aspects of the food label, such as nutrition information panels and front-of-pack systems such as the health star ratings, combined with limited knowledge of the regulation of claims, exacerbated mistrust and skepticism. Although participants reported awareness and recognition of nutrition content and health claims, habits and price were the most salient factors influencing food purchasing decisions.

It is important to analyze the findings from previous systematic reviews and meta-analyses, as these approaches reduce selection and interpretation biases by critically appraising primary studies in a systematic manner. In particular, meta-analyses enable the statistical combination of results, increasing analytical power and allowing more precise effect size estimates, as well as the exploration of heterogeneity across studies. As these publications were looking for consolidated data, Teoh et al. [87] evaluated the factors impacting consumers’ decisions (such as health benefits, advice from professionals, cost, and accessibility). In their study, consumer knowledge and the benefits perceptions present a key role in consumer behavior, but they reinforce the limitations of this conclusion. Additionally, the results indicate variability according to population and the type of nutrient. In another systematic review from Ballco and Gracia [45], the authors highlighted the limited role of the relationship between health claims and consumer behavior. Their results presented heterogeneity and inconsistencies depending on the claim type and consumer profile. According to the authors, taste was the most important intrinsic characteristic, and consumers are not willing to sacrifice the pleasure of sensory function for health benefits. Perceived healthiness, understanding of the claims, liking, and use were factors that affected consumers’ personal processes in purchasing food with nutritional claims and health claims [45]. The systematic review and meta-analysis conducted by Baker et al. [44] assessed how consumers’ level of knowledge about functional foods positively impacted their purchase intention, acceptance, willingness to pay, and consumption-related behaviors. In general, greater knowledge about functional foods was significantly associated with greater acceptance and intention to consume these products. In addition, more informed consumers tend to perceive functional foods as more beneficial to health and demonstrate less skepticism. However, the review also identified important variations between studies, suggesting that the type of knowledge (e.g., technical versus practical knowledge) and the way this knowledge is measured may influence the results. Their meta-analysis demonstrated a moderate positive overall effect, reinforcing the idea that educational and informational strategies can be effective in promoting the acceptance of functional foods in the market. The article concludes by recommending that future marketing and communication efforts not only inform consumers about the presence of functional components but also educate in a clear, practical, and reliable manner to improve the adoption of these products [44]. The findings of the present narrative review are consistent with previous systematic reviews and meta-analyses, indicating that the relationship between health claims and consumer behavior is complex and context-dependent, with limited quantitative synthesis available. It is therefore not possible to establish a clear and isolated influence of the health claim phrase on individual behavior. The role of health claims on consumer behavior is observed in most parts of the literature, but the positive attitudes that influence consumer choice are not only dependent on the presence of health claims in the labeling of food products.

Regarding sociodemographic profile, individuals with higher income and education tended to demonstrate greater acceptance and attention to functional claims. In terms of age, young adults were more receptive to these claims, while older individuals showed particular interest in claims related to disease prevention. Regarding gender, women showed greater willingness to balance taste and health attributes when choosing foods [69]. Chattaraman et al. [39], interestingly, investigated how social distance (thinking about oneself versus thinking about children) could modulate the claims’ influence. When consumers chose food for their children, their attitudes were more positive towards health claims. Marketing communication strategies and public policies may benefit from adjusting the type of claim based on the consumer’s context (for self or for children). Finally, despite the limited effectiveness of claims in directly driving purchase intention, attitudes toward products may be more sensitively shaped by these messages, especially when well-aligned with the consumer’s psychological context [39].

The health claim phrase construction seems to show importance in the context of its relationship with consumers. Hodgkins et al. [85] highlight that the level of complexity of health claims plays a crucial role in how consumers interpret these messages and make purchasing decisions. Overly technical or complex claims, which require greater nutritional interpretation skills, tend to be poorly understood by most consumers, particularly among those with low levels of health literacy. In contrast, simpler and more direct claims are more effective in conveying the intended message and positively influencing food choices. Studies emphasize that the balance between simplicity of language and scientific accuracy is essential: claims need to communicate the benefit in a manner to facilitate understanding and allow quick decisions in the purchasing environment, without sacrificing the accuracy of the information conveyed. When claims are formulated in a simple way, consumers are more likely to correctly interpret the nutritional or health benefit advertised, especially those with less technical knowledge. These references also recommend that public policies and regulations encourage the standardization of claims in simple, direct, and consistent formats to reduce the cognitive load on the consumer at the time of decision. In addition, clear and low-complexity claims can minimize the risk of exaggerated or misinterpretation, a phenomenon often observed when messages are formulated in a vague or ambiguous manner [85,88]. Other studies corroborate this suggestion, exploring the communication tools [57,59].

Studies have indicated that the use of decorative images related to health on packaging can induce false memories in consumers, leading them to believe that they had read claims that were not present [46]. Another relevant aspect identified was that emotional eating behavior can reduce the willingness to pay for foods with health claims [94]. The review conducted by Baker et al. [49] concluded that consumer acceptance of functional foods is a complex and multifactorial phenomenon, influenced by a combination of product characteristics, sociodemographic, psychological, behavioral, and physical factors.

Despite the growing body of literature examining health claims, a critical analysis of the evidence reveals several recurring limitations. Most studies rely on self-reported questionaries and tests without observation of the real behavior measures (for example: real observed purchase). The questionaries and tests focused on theoretical measures of attitudes, intentions, or perceived understanding, which may not accurately reflect actual purchasing behavior. Moreover, considerable heterogeneity exists in study designs, population representativity, and outcome measures, which are the limitations that justify that the observed associations between health claims and consumer responses should be interpreted with caution.

### 4.2. Elaboration Likelihood Model and Theory of Planned Behavior

Interpretations of how consumers process health-related information on food labels can be supported by established behavioral theories, particularly the Elaboration Likelihood Model (ELM) [96] and the Theory of Planned Behavior (TPB) [97]. According to the ELM, consumers may process information through a central route, involving careful evaluation of claim content and scientific justification, or through a peripheral route, relying on heuristics such as claim framing, symbols, or source credibility. This distinction is particularly relevant in the context of health claims, as consumers’ motivation, ability, and prior knowledge influence how claims are interpreted and used in decision-making. Complementarily, the TPB explains food choice behavior as the result of behavioral intentions shaped by attitudes, subjective norms, and perceived behavioral control. In the context of health and functional claims, attitudes may be influenced by perceived health benefits, subjective norms by trust in experts or social endorsement, and perceived behavioral control by factors such as accessibility, price, and clarity of information. Together, these frameworks provide a conceptual basis to interpret the diverse consumer responses reported in the literature.

Steinhauser et al. [84,89], with the use of eye tracking, observed that consumers with higher knowledge of nutrition and higher health motivation looked at nutrition and health claims to a greater extent when making a purchase decision compared to other consumers. This is aligned with ELM as knowledge and motivation led to a different elaboration. Furthermore, behavioral characteristics, such as adopting healthy lifestyles and previous familiarity with functional foods, increase the propensity to consume these products. Physical factors, such as the presence of health problems or a high body mass index, were also associated with greater acceptance of functional foods, reflecting the search for alternatives that promote improved health [44].

**Figure 8:**
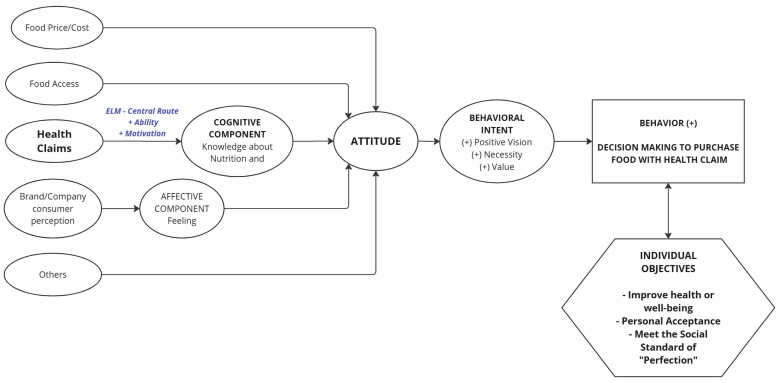
Framework Health Claims in Consumer Behavior. Source: Authors.

By explicitly linking the empirical findings to ELM and TPB, this review highlights that the effectiveness of health claims depends both on the presence of clear information and how consumers understand it, as well as which behavioral determinants are activated. This theoretical integration helps clarify why health claims do not exert uniform effects and underscores the need for future research to adopt more theory-driven designs. The framework adopted in this work to model consumers’ behavior involving functional foods with health claims is demonstrated in Figure 8.

The communication of health claims has a role on consumer’s behavior, but this role is not isolated. It is an additional factor of food choice or decision, together with other well-recognized factors such as price, brand, and food preferences. Health claims can influence the consumer’s cognitive component through a central route (ELM), increasing the ability and motivation of consumers to understand the health benefits of the food product. This central route persuasion could elicit memories from their previous knowledge of nutrition and still offer new information, creating a positive attitude toward the product. This positive attitude can influence the consumer’s behavioral intention due to an increase in value, higher necessity perception, leading them to a behavioral action in favor of the food. The Theory of Planned Behavior explains that the presence of a proportional and well-regulated functional claim could be relevant to help consumers make more informed and health-conscious purchasing decisions, as indicated by some studies [44,98,99].

The findings of our narrative review and bibliometric analysis should be interpreted in light of certain limitations, including the selection of databases and language restrictions, which may have influenced the scope of the retrieved literature. Moreover, the heterogeneity of study designs and methodological approaches precluded quantitative synthesis and may have affected the comparability of results. Another limitation of this study is the potential inability to fully account for subjective elements in the investigation of decision-making processes related to food purchases. Factors such as impulsive choice behavior and the influence of emotions, which can significantly impact food choices and consumer behavior, may not be adequately captured or quantified in the study design. As a result, our findings may not fully reflect the complexity of decision-making in real-world food purchasing scenarios.

## 5. Conclusions

We demonstrate that the communication of health claims is a vast field of study, with heterogeneous results regarding their effects on consumer behavior. Despite several studies indicating a positive role of health claims on consumers’ behavior, particularly by enhancing perceived value, purchase intention, and willingness to pay, the effectiveness of these claims is not uniform and depends on multiple individual and contextual factors [21,22,23,24]. These factors include product characteristics, price, educational level, and the type of claim presented. Additionally, considering the articles included in this narrative review, a critical analysis of the current evidence reveals several recurring limitations, such as qualitative studies, no representative population, and diverse design protocols.

From a practical perspective, these findings have important implications for policymakers, regulators, and the food industry, highlighting the need for clear, credible, and consumer-oriented communication strategies that account for audience heterogeneity. Well-designed functional claims may support healthier food choices when aligned with consumers’ cognitive abilities and motivational states.

Future research should focus on experimentally testing the effectiveness of different types of health claims across consumer segments, product categories, and cultural contexts. In addition, further integration of behavioral theories, such as the ELM and TPB, with regulatory and marketing perspectives may advance the development of evidence-based guidelines for nutritional labeling and health communication in the food sector.

## Figures and Tables

**Table 1:** Descriptive data based on databases compilation.

Description	Web of Science	Scopus	PubMed
Articles	423	420	44
Articles (year of higher production)	60 (2020)	56 (2020)	7 (2022)
Most intense period of publication	2018–2020	2014–2020	2020–2022

**Table 2:** Web of Science Database: Highest Cited Authors.

Number of Citations	Indication in VOSviewer	References
297	Miller	[27]
282	Williams	[29]
267	Chandon	[30]
232	Ikonen	[31]
214	Cohen	[32]
196	Verbeke	[28]
182	De Jong	[33]

**Table 3:** Studies Characteristics and Health Claims Role on Consumer Behavior: Attitudes, Perceptions, Food Values, Willingness to Pay.

Reference	Population (n)	Method	Country	Was the Role of Health Claims on Consumer Behavior Observed?
Mohammad et al. [34]	313	Questionary	Hungary	Yes
Safraid et al. [35]	522	Questionary	Brazil	Yes
Toorani et al. [36]	536	Questionary	Iran	Yes
Reitano et al. [21]	333	Questionary	Italy	Yes
Kowalska et al. [10]	784	Questionary	Poland	Yes
Huang et al. [37]	630	Questionary	China	Yes
Collins & Lalor [38]	24	Focus Group + Questionary	Ireland	Yes
Jahdav et al. [22]	400	Review + Questionary	India	Yes
Chattaraman et al. [39]	171	Questionary	US	Yes
Grujić & Grujčić [40]	720	Questionary	BH	Yes
Dias et al. [41]	303	Questionary	Sri Lanka	Yes
Bou Fakhreddine & Sánchez [42]	207	Questionary + Sensorial	Spain	Yes
Rai et al. [23]	-	Review	Thailand	Yes
Neale & Tapsell [43]	-	Review	Australia	Yes
Baker et al., 2022 [44]	-	Systematic Rev/Meta-Analysis	US	Yes
Tonnesen et al. [19]	1494	Questionary	Denmark	Yes
Ballco & Gracia [45]	-	Systematic Review	Spain	No–Limited relationship
Delivett et al. [46]	60	Memory Test: label images	UK	Yes
Tian et al. [47]	2379	Test: leaflets + videos	China	Yes
Wu et al. [48]	1046	Questionary	Taiwan	Yes
Baker et al., 2022 [49]	-	Review	US	Yes
Kosicka-Gebska et al. [50]	1034	Questionary	Poland	Yes
Stuthridge et al. [20]	49	Focus Group + Questionary	NZ	No–Limited relationship
Teoh et al. [51]	111	Questionary	Malaysia	Yes
Nystrand & Olsen [52]	810	Questionary	Norway	Yes
Nystrand et al. [53]	810	Questionary	Norway	Yes
Wang & Chu et al. [54]	398	Questionary	Taiwan	Yes
Duarte et al. [55]	477	Review + Questionary	Portugal	Yes
Topolska et al. [56]	-	Review	Poland	Yes
Schifferstein et al. [57]	-	Review	N	Yes
Arfaoui et al. [58]	722	Questionary	SA	Yes
Fatkullin et al. [59]	721	Questionary	Russia	Yes
Rizwana et al. [60]	301	Questionary	India	Yes
Kandyliari et al. [61]	949	In vitro + Questionary	Greece	Yes
Plasek et al. [62]	633	Questionary + prod. images	Hungary	No–Limited relationship
Bryla [63]	1051	Questionary	Poland	Yes
Di Vita et al. [64]	767	Questionary	Italy	Yes
Shammakh et al. [65]	385	Questionary + prod. image	Malaysia	Yes
Papp-Bata & Szakaly [66]	16	Focus Group	Hungary	Yes
Vorage et al. [67]	350	Questionary	Australia	Yes
Nguyen et al. [68]	596	Questionary	Vietnan	Yes
Szakos et al. [69]	1002	Questionary	Hungary	Yes
Gonzalez-Diaz et al. [70]	191	Questionary	Spain	Yes
Costa & Strehlau [71]	44	Focus Group	Brazil	Yes
Plasek et al. [72]	-	Review	Hungary	Yes
Menozzi et al. [73]	2059	Questionary	F, G, I, S, UK	Yes
Banjari et al. [74]	452	Market + Questionary	Croatia	Yes
Nystrand & Olsen [75]	810	Questionary	Norway	Yes
Ballco et al. [76]	218	Track-Eye + Sensorial	Spain	Yes
Nguyen [77]	260	Questionary	Spain	Yes
Guiné et al. [2]	-	Review	Portugal	Yes
Biondi & Camanzi [78]	1250	Questionary	Italy	Yes
Ali & Ali [79]	218	Questionary	India	Yes
Franco-Arellano et al. [80]	1997	Questionary	Canada	Yes
Klopčič et al. [81]	45	Focus Group	Slovenia	Yes
Bakti et al. [82]	123	Questionary	Indonesia	Yes
Theben et al. [83]	300	Questionary	Spain	No–Limited relationship
Steinhauser et al. [84]	156	Questionary + Track-Eye	Denmark	Yes
Hodgkins et al. [85]	100	Questionary	G, N, S, Sp, UK	Yes
Ballco & Magistris [24]	218	Questionary	Spain	Yes
Temesi et al. [86]	1016	Questionary	Denmark	Yes
Teoh et al. [87]	-	Systematic Review	Malaysia	Yes
Annunziata & Mariani [88]	508	Questionary	Italy	Yes
Steinhauser et al. [89]	156	Questionary + Track-Eye	Germany	Yes
Samoggia & Riedel [90]	250	Questionary	Italy	Yes
Lusk [91]	1250	Questionary	US	Yes
Benson et al. [92]	78	Focus Group	Ireland	Yes
Szakaly et al. [93]	500	Questionary	Hungary	Yes
Lopez-Galan & de-Magistris [94]	306	Questionary	Spain	Yes
Ballco et al. [95]	100	Questionary + Track-Eye	Spain	Yes
Viscecchia et al. [18]	601	Questionary	Italy	No–Limited relationship

Abbreviations: BH: Bosnia Herzegovina; US: United States; SA: Saudi Arabia; NZ: New Zealand; F: France; I: Italy; G: Germany; N: Netherlands; S: Slovenia; Sp: Spain; UK: United Kingdom.

**Table 4:** Health Claims Phrases’ relationship with consumer behavior: attitudes, perceptions, willingness to pay, and perceived food value.

Reference	Food Category	Evaluation of Consumer’s Behavior Towards Functional Foods	Relationship Observed Between Health Claims and Consumer Behavior	Wording & Phrase: Health Claims Evaluated
[39]	Whole-grain wheat Low-fat dairy	**Attitude and Purchase Intentions:** questionary using product photo prototypes.	Yes	“May Reduce the Risk of Heart Disease”; “May Reduce the Risk of Osteoporosis”.
[42]	Extra Virgin Olive Oil	**Purchase intention:** questionary considering an informed scenario with and without a health claim, in addition to sensorial analysis.	Yes	“Olive oil polyphenols contribute to the protection of blood lipids from oxidative stress”.
[19]	General Foods Healthy (Broccoli, Strawberry) Unhealthy (Chips, Cake, Sausages) and Health-neutral (Fruit-yogurt, ham, vegetarian cold cuts)	**Choice Task:** questionnaire where respondents were shown eight different products with and without Nutrition and Health Claims. The products varied in terms of health and taste perception.	Yes	“Broccoli has a high content of Vitamin K. Vitamin K contributes to the maintenance of normal bone”. “Strawberries have a high content of vitamin C. Vitamin C contributes to a normal functioning nervous system.”
[20]	Diverse Functional Foods	**Attitude:** Qualitative study. Analysis using inductive coding, with development of five themes: (1) aware of claims but did not use, (2) mistrust and skepticism, (3) confusion and misinterpretation, (4) using claims to guide food choice, and (5) not all claims are equal.	Depending on consumer profile	“Cholesterol Lowering” “Actively Lowers Cholesterol”
[58]	Functional Foods	**Consumer knowledge and use of health claims:** Questionary applied, and study highlights the need for more education and public awareness programs to enhance consumer knowledge and use of the nutrition facts label and health claims, and consequently lead to healthy dietary choices.	Yes	Health claims relationship questions: “Vitamin D deficiency and Osteoporosis”; “High fat intake and heart diseases; “High sodium intake and hypertension”; “High fiber intake and Diabetes Mellitus”
[62]	Functional Foods (Smoothie product)	**Perception of health impacts:** questionary examined the effects of 6 attributes: claims related to ingredients, organic origin, health claims, shape of packaging, color of packaging, and domestic origin. In the order of importance, health claims/nutritional claims are only the fifth of the six elements, and only the nutritional claim showed significant effect; the tested health claim did not.	Limited results to health claims (extrinsic characteristics—blue color and organic origin—have greatest effect on consumers’ beliefs).	“Protein contributes to the maintenance of normal bones”.
[63]	General Foods	**Consumer behavior:** the study identified selected predictors of food labels in consumer behavior and food purchases. The importance of the information about the content of fat and that about the health effects of consuming a food product were significant predictors of three types of food label use.	Yes	Health information included in the questions: “Lowering cholesterol”; “Reducing the risk of heart diseases”; “Strengthening bones”; “Impact on the digestive system”; “Reducing tiredness and fatigue”; “Maintaining proper vision”; “Proper development of children”; “Proper functioning of the heart”.
[64]	Extra Virgin Olive Oil	**Preferences:** different degrees of individual knowledge act as distinctive drivers in influencing the health perception of olive oil consumers. The findings reveal that label information is only important for uninformed consumers, since it is positively correlated with a healthy product. This could indicate that less informed consumers use labels as a quality signal to detect information about the health components of olive oil.	Yes	Health properties included in the questionary: “Anti-inflammatory activities”; “Positive action on the immune system”; “Prevention of cardiovascular disease”.
[67]	Functional Foods	**Attitude and Food Choice:** The study analyzed some predictors for attitude and choice. Of the eight predictors, three were statistically significant: living situation, natural content, and health. Findings highlight that when targeting emerging adults, functional food companies could benefit from promoting the natural and health properties of their products.	Yes	Questionary included some claim examples as: “margarine which can lower cholesterol”; “yogurt drink with probiotics”.
[73]	Fish Products (trout, herring, salmon, sea bass, sea bream, cod, and pangasius)	**Preferences and Willingness to pay:** The questionaries results showed positive perspectives for a sustainability label, and nutrition and health claims, with high heterogeneity across species and countries. The results may also suggest the need to implement homogeneous strategies within EU countries, for educating consumers about the product labeling and the different claims and certifications which can be found on the pack, and about the tangible benefits to consumers related to health and sustainability labels.	Yes	“Product high in omega-3 fatty acids, which contribute to the maintenance of normal function of the heart and normal blood pressure, with the following condition of use: The beneficial effect is obtained with a daily intake of 250 mg of omega-3 fatty acids. Such an amount can be consumed as part of a balanced diet”.
[74]	Functional Dairy (milk, yogurt, and cheese)	**Attitudes:** Consumers’ awareness improved over time, especially regarding probiotics. Consumers are more likely to attribute a particular health effect to a functional product, rather than generally describing them as “good for health”, but also more people associate functional foods with organic products. These results imply the need for manufacturers to strengthen and better target communication strategies for not only the new products, but the existing ones as well.	Yes	Questionary included a specific question: “How would you define what functional foods are? Possible answers: (i) products with a positive effect on health; (ii) products that prevent diseases; (iii) products that will lower blood cholesterol.”
[76]	Functional Foods (Yogurt)	**Consumer Attention:** The results suggest that there is a relationship between the highly valued nutrition claims and Health claims from the stated preferences and visual attention in terms of fixation count. This relationship affirms that the final product selection is based not only on the type of labeling on the package but also on the visual attention that consumers pay to it.	Yes	“Calcium is necessary for maintaining bones under normal conditions”; “Vitamin B6 helps your defenses and reduces fatigue”.
[81]	Breakfast Cereal	**Preference:** The survey demonstrated that consumers in general are moderately doubtful of nutrition and health claims. Conjoint analysis showed that when Slovenians choose their breakfast cereals, nutrition and health claims are more important than whether visual images are present.	Yes	“For a healthy heart” (general non-specific health claim) and “Cholesterol-lowering” (a more specific claim).
[82]	Functional Foods	**Purchase:** Attitude and subjective norm influence the purchase intention of the young consumers to buy functional foods.	Yes	Questionary included specific questions for Attitude: AT1: I like functional food that can prevent hypertension; AT4: It is interesting to consume functional food that can prevent hypertension.
[84]	Orange Juice Milk Chocolate	**Purchase behavior:** The findings indicate that each claim was noticed by at least 85% of the participants, and health claims were looked at longer than nutrition or taste claims. When compared to other participants, the longer a participant looked at a specific claim, the more likely the participant was to purchase the respective product.	Yes	Orange Juice: “Vitamin C contributes to the normal function of the immune system”; Milk Chocolate: “Calcium is needed for the maintenance of normal bones”.
[85]	-	**Perception:** consumers do not consciously differentiate between nutrition claims and health claims in the same way regulatory experts do. Additionally, a consumer-derived typology of health-related claims was identified, based on three main dimensions: (1) familiarity with the nutrient, substance, or food mentioned in the claim; (2) statement type in terms of simplicity/complexity; and (3) relevance of the claim, either personally or for a specific population group.	Yes	Examples of the Health Claims evaluated: “Calcium is needed for the maintenance of normal bones”; “Vitamin B12 contributes to normal homocysteine metabolism”; “Iron contributes to the normal cognitive development of children”; “Zinc contributes to normal cognitive function”; “Vitamin D contributes to the maintenance of normal muscle function”; “DHA contributes to normal brain function”; “Pantothenic acid contributes to normal mental performance”; “Taurine contributes to normal eye/vision development in children”; “Reduced risk of heart disease”.
[24]	Yogurt	**Purchase behavior:** Results suggest that consumers positively valued most claims, however, the valuation was heterogeneous, and three consumer segments were identified: “health-claims oriented”, “nutritional-and health-claim oriented”, and “indifferent”.	Depending on consumer profile	“Reducing consumption of saturated fat contributes to the maintenance of normal blood cholesterol levels”; “Consumption of food containing sweeteners instead of sugar induces lower blood glucose”; “Fiber contributes to an acceleration of intestinal transit”; “Fiber contributes to an increase in fecal bulk”; “With vitamin B6 that helps your defenses and reduces fatigue”; “Vitamin B6 contributes to the normal functioning of the nervous system”; “Calcium is necessary for maintaining bones under normal conditions”; “Calcium contributes to normal muscle function”.
[89]	Orange Juice	**Consumer knowledge:** The lower the price and the higher the perceived healthiness and tastiness of the product further it heightened its likelihood of being purchased. Interestingly, consumers with higher nutrition knowledge and/or higher health motivation looked longer at the nutrition and health claims; however, these consumer characteristics did not show an effect on the purchase decision.	Yes	“Vitamin C contributes to the normal function of the immune system”.
[90]	Coffee	**Perception and Purchase:** Consumers drink coffee for its energetic and therapeutic effects. Coffee consumption is still price-driven, but consumers are interested in purchasing coffee with associated health claims.	Yes	Health Claims in the Questionary: Awakening and attention Physical energy Digestion Against headache Increase blood pressure
[18]	Mozzarella cheese	**Consumer’s trade off:** consumers are unfamiliar with functional foods; analysis reveals distinct consumer groups and a higher WTP for health and disease-risk reduction claims than for nutrition claims alone.	Depending on consumer profile	“Contributes to the maintenance of normal blood cholesterol levels”; “Helps to reduce cardiovascular disease risk”.

## Data Availability

Dataset available on request from the authors.

## Abbreviations

The following abbreviations are used in this manuscript:

WHO	World Health Organization
FOSHU	Foods for Specified Health Uses
WTP	Willing to Pay
ELM	Elaboration Likelihood Model
TPB	Theory of Planned Behavior
